# Association of Blood Cadmium with Cardiovascular Disease in Korea: From the Korea National Health and Nutrition Examination Survey 2008–2013 and 2016

**DOI:** 10.3390/ijerph17176288

**Published:** 2020-08-28

**Authors:** Jihyun Jeong, Sang-moon Yun, Minkyeong Kim, Young Ho Koh

**Affiliations:** Division of Brain Diseases, Center for Biomedical Sciences, Korea National Institute of Health, 187 Osongsaengmyeong2-ro, Osong-eup, Heungdeok-gu, Cheongju-si, Chungcheongbuk-do 28159, Korea; jhjeong1107@gmail.com (J.J.); ysm0304.0304@gmail.com (S.-m.Y.); kminkyeong94@gmail.com (M.K.)

**Keywords:** cadmium, stroke, hypertension, non-elderly population, KNHANES

## Abstract

Cardiovascular disease (CVD) is the leading cause of death globally, although the mortality rate has declined with improved technology and risk factor control. The incidence rate of stroke, one of the CVDs, is increasing in young adults, whereas it is decreasing in the elderly. The risk factors for CVD may differ between young adults and the elderly. Previous studies have suggested that cadmium was a potential CVD risk factor in the overall and middle-aged to elderly populations. We assessed the associations between cadmium and CVD events in the Korean population aged 20–59 years using the 2008–2013 and 2016 Korea National Health and Nutrition Examination Survey (KNHANES), a population-based cross-sectional study. Among 10,626 participants aged 20–59 years, those with high blood cadmium (BCd) level (>1.874 µg/L, 90th percentile) were higher associated with stroke and hypertension (stroke: odds ratio (OR), 2.39; 95% confidence interval (CI), 1.03–5.56; hypertension: OR, 1.46; 95% CI, 1.20–1.76). The strongest association between high blood cadmium concentrations and hypertension was among current smokers. Ischemic heart disease (IHD) was not associated with high blood cadmium level. These findings suggest that high blood cadmium levels may be associated with prevalent stroke and hypertension in the Korean population under 60 years of age.

## 1. Introduction

Cardiovascular disease (CVD) describes disorders of heart and blood vessel including cerebrovascular disease, coronary heart disease such as stroke, and ischemic heart disease (IHD) [1]. CVD is a global health problem [2] and the leading cause of death worldwide [1]. Although, the mortality rate of CVD has declined due to controlling risk factors and technology advances [3], it was slow among the young aged population because of the increasing incidence of CVD in young adults [3]. Mortality due to stroke, one of the CVDs, is also declining; however, the absolute number of stroke patients is increasing [4]. The incidence rate of stroke is increasing among non-elderly aged <45 years [5] or <55 years [6], whereas it is decreasing in the elderly [5]. In addition, the incidence of hypertension, which is associated with the risk of CVD, is increasing in young adults aged 18–35 years [7]. However, few studies have assessed the risk factors for CVD among non-elderly individuals because CVD mainly develops in the elderly [8,9]. More studies are needed to prevent and manage the risk of CVD for the non-elderly population.

The risk factors for CVD in young patients may not be the same as those in elderly patients [10,11,12,13]. The Helsinki Registry, the first large study of young adults under 50 years of age with ischemic stroke (IS), reported that young IS patients had less related standard risk factors for stroke such as hypertension, hyperlipidemia, and diabetes compared to older patients with acute ischemic stroke (AIS) [14]. In Asia, a hospital-based study in Taiwan reported that young stroke patients (<65 years of age) were less likely to have traditional stroke risk factors such as atrial fibrillation (AF) and history of stroke. Instead, they had high proportions of history of smoking, hypercholesterolemia, hypertriglyceridemia, and high body mass index (BMI) [10]. Recently, the risk factors for stroke were reported according to age in Korea. Smoking was a strong risk factor for stroke in young adults (<45 years), while hypertension and diabetes were related stroke events in middle-aged adults (45–64 years) and AF was a major risk factor for stroke in older populations (≥75 years) [15]. The risk factors for stroke among young adults are not the traditional factors. Thus, there is a need to identify the risk factors for stroke in young patients.

Exposures to environmental toxic metals have harmful effects on humans [16,17,18]. Several metals such as arsenic, lead, cadmium, and copper have been related to an increased risk of CVD and coronary heart disease [19]. Among these metals, cadmium level is associated with not only aging [20] but also smoking [21,22], food [23], and dust [23]. Smokers had a 1.9 times higher mean level of BCd than non-smokers [24], because tobacco plants contain cadmium [25]. In Korea, the proportion of cigarette smokers was higher in the non-elderly (<60 years) and middle-aged populations than the elderly (≥60 years) during the past decade [26]. Thus, cadmium accumulation from smoking may affect the non-elderly population to a greater degree. In addition, a recent study in Korea has suggested that smoking was a major stroke risk factor in young adults (<45 years) [15]. These results led us to investigate the relationship between Cd and CVD including stroke among non-elderly individuals. Absorbed cadmium in the human body increases the risk of CVD through endothelial cell death, oxidative stress, smooth muscle cell accumulation, and vascular inflammation [27,28].

Previous studies have reported cadmium as a predictive risk factor for CVD. A meta-analysis suggested that cadmium level was associated with the risks of CVD, coronary heart disease (CHD), and peripheral arterial disease (PAD) but not with stroke risk among overall adults (≥20 years) and the middle-aged to elderly populations [29]. However, a heterogeneity is in the association between cadmium level and risks of CVD, CHD, and stroke in this meta-analysis, respectively [29]. It reflects inconsistent results regarding Cd–CVD in various studies. Hypertension and/or blood pressure were also related to cadmium exposure. It has been suggested that cadmium levels were related to a higher risk of hypertension in adults ≥20 years of age in Korea [30,31,32,33]. However, nearly all studies on the association between cadmium levels and CVD were conducted in adults ≥20 years of age or older than middle-aged. Few studies have explored CVD risk factors among young adults even though the incidence of CVD is increasing in young adults [3].

We aimed to determine whether blood cadmium (BCd) level is associated with the risk of prevalent CVD such as stroke, hypertension, and IHD among participants under 60 years of age in the 2008–2013 and 2016 Korea National Health and Nutrition Examination Survey (KNHANES).

## 2. Subjects and Methods

### 2.1. Study Population

**Figure 1:**
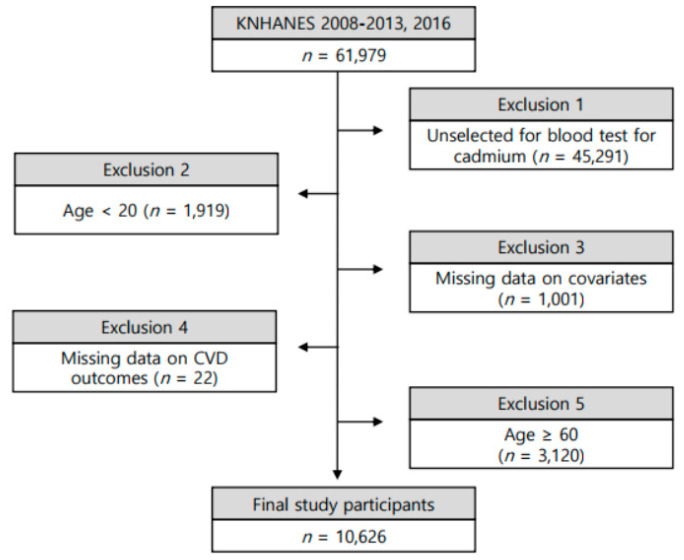
Schematic diagram depicting study population. KNHANES: Korea National Health and Nutrition Examination Survey.

We used data from the 2008–2013 and 2016 KNHANES, conducted by the Korean Ministry of Health and Welfare. This is a representative community-based cross-sectional survey in Korea. It examines approximately 10,000 individuals each year and collects information on socioeconomic status, health-related behaviors, anthropometric measures, biochemical information, clinical information, and dietary intakes through three component surveys: health interview, health examination, and nutrition survey [34]. Detailed information about the protocol and design of the KNHANES has been provided previously [34]. The heavy metals including cadmium were measured partially for individuals randomly in the KNHANES. The 2008–2013 and 2016 KNHANES included 16,688 participants with complete health examinations including BCd measurements. We excluded those under 20 years of age, those over 60 years of age, and those with missing information (Figure 1).

All participants completed an informed consent form and the KNHANES survey was approved by the Institutional Review Board (IRB) of the Korea Centers for Disease Control and Prevention (KCDC) annually, which allowed people to use the KNHANES data formally and ethically (IRB numbers: 2008-04EXP-01-C, 2009-01CON-03-2C, 2010-02CON-21-C, 2011-02CON-06-C, 2012-01EXP-01-2C, 2013-07CON-03-4C). The IRB approval was not required for KNHANES 2016 as the purpose of this survey was welfare. We approved data acquisition in the present study by the IRB of the KCDC under approval number 2017-05-06-2C-A, 29 July 2019, and obtained data from the website (https://knhanes.cdc.go.kr).

### 2.2. Covariate Variables and CVD Outcomes

Sociodemographic data (age, sex, education level (less than high school diploma, high school diploma, college diploma or higher), house income level (quartile) were collected using the face-to-face interview method. Smoking status (never, former, current) and frequency of alcohol consumption per week (0, 1, 2 or 3, ≥4) were collected using a self-reported questionnaire. Participants responding “daily smoking” or “occasional smoking” to these questions were categorized as current smokers. Those who answered “smoking in the past but not smoking now” were categorized as former smokers. Never-smokers were classified as those who did not answer using current smoker and former smoker responses. Physical activity including walking was collected through a face-to-face interview. Regular walking defined participants who had walked at least 30 min at a time and ≥5 days a week during the last seven days [35].

Diabetes mellitus (DM) was defined as a fasting plasma glucose concentration ≥126 mg/dL, use of oral anti-diabetes medication or injected insulin, or diagnosis of diabetes from a physician [36]. Impaired fasting glucose (IFG) was diagnosed based on fasting plasma glucose levels ≥100 and ≤125 mg/dL [36]. Obesity was defined as BMI ≥25 kg/m^2^, which was calculated as the body weight in kilograms divided by the height in meters squared. Low-density lipoprotein cholesterol (LDL-c) was calculated using the Friedewald formula with the following levels of total cholesterol (TC), triglycerides (TGs), and high-density lipoprotein cholesterol (HDL-c):$\;LDL-c\;\left(mg/dL\right)=TC\;\left(mg/dL\right)-HDL-c\;\left(mg/dL\right)-TG\left(mg/dL\right)/5$. Estimated glomerular filtration rate (eGFR) was calculated using the Modification of Diet in Renal Disease (MDRD) equation with the serum creatinine level; $eGFR\;\left(mL/min/1.73m^{2}\right)=186\times serum\;creatinine{\left(mg/dL\right)}^{-1.154}\times Age^{-0.203}\times \left(0.742\;if\;female\right)$. 

Stroke and IHD were diagnosed according to affirmative responses to questions on the self-reported questionnaire asking if the subject had been diagnosed with this disease by a doctor. Participants responding “Yes” to these questions were categorized as having stroke and IHD. Blood pressure was measured three times on the subject’s right arm using an appropriately sized arm cuff and mercury sphygmomanometer (Baumanometer; WA Baum Co., New York, NY, USA). It was measured in a seated position for at least 5 min and the final BP was the mean value of the second and third measurements [37]. Hypertension was defined as a systolic blood pressure (SBP) ≥140 mmHg or a diastolic blood pressure (DBP) ≥90 mmHg, or use of anti-hypertensive medications, or diagnosis from a doctor. The pre-hypertension criteria were 120 ≤ SBP < 140 mmHg, or 80 ≤ DBP < 90 mmHg [38].

### 2.3. Determination of BCd Levels

The heavy metals including blood cadmium were collected into standard evacuated tubes containing sodium heparin. The determination of blood cadmium was measured by graphite furnace atomic absorption spectrometry (AAnalyst 600, PerkinElmer, Turku, Finland) with Zeeman-effect background correction [39,40]. The cadmium concentration was measured by the Neodin Medical Institute, a laboratory certified by the Korean Ministry of Health and Welfare. The coefficients of variation of the blood cadmium measurement were within 0.95~4.82%. The limit of detection (LOD) for blood cadmium level was 0.056 μg/L in the present study [41,42]. There were no samples below the LOD level. High BCd level was defined as a BCd concentration >1.874 μg/L, which corresponded to the 90th percentile in the present study.

### 2.4. Statistical Analysis

We conducted statistical analysis using SAS (version 9.4; SAS Institute Inc., Cary, NC, USA). In the complex sampling study, sampling weights were used in all analyses. The log-transformed BCd concentrations were used to calculate the geometric means (GMs). The differences’ mean level of variables between non-high BCd and high BCd groups was assessed by survey t-tests. Demographic and clinical differences between the non-high and high BCd groups were assessed using Rao–Scott chi-square tests. The *p*-values for the trends were calculated by survey regression tests after adjusting for age, sex, current smoking status, education level, income level, alcohol consumption, BMI, glucose level, systolic blood pressure, survey year, and regular walking. The odds ratios (ORs) and 95% confidence intervals (CIs) for stroke, hypertension, and IHD were analyzed using a survey logistic regression model after adjusting for all covariates, which were used in the survey regression test and further adjusted for eGFR. We conducted a stratification analysis according to smoking status for hypertension prevalence to evaluate the smoking effect on the association between high BCd and prevalent hypertension risk.

## 3. Results

### 3.1. Baseline Characteristics and BCd Concentrations

A total of 10,626 adults (5081 (50.4%) men and 5545 (49.6%) women) were under 60 years of age and 3120 (1487 (43.0%) men and 1633 (57.0%) women) were ≥60 years of age. Among adults <60 years of age, the prevalence of stroke, hypertension, and IHD was 60 (0.46%), 1930 (18.3%), and 80 (0.75%), respectively. Among those ≥60 years of age, the prevalence of stroke, hypertension, and IHD was 150 (4.6%), 1773 (57.8%), and 184 (5.7%), respectively. The general characteristics of the present study participants are given in Table 1. The mean age was 40 years among all participants, with a higher mean age in the high BCd group than that in the non-high BCd group (47 vs. 39 years, *p* < 0.0001). In the high BCd group, the proportion of women was higher than men. Overall, 60 (weighted prevalence = 0.5%), 1930 (18%), and 80 (0.8%) participants had stroke, hypertension, and IHD, respectively. The proportion of current smokers was significantly higher in the high BCd group than that in the non-high BCd group (*p* < 0.0001). There were significant differences between the non-high and high BCd groups in age, sex, smoking status, daily smoking amount (the number of cigarettes per day), education level, income level, alcohol consumption, SBP, DBP, BMI, glucose level, eGFR, diabetes status, regular walking, stroke, and hypertension prevalence.

Table 2 presents the GMs of the BCd levels in the non-high and high BCd groups. The mean BCd levels were 0.89, 0.80, and 2.40 μg/L overall and in the non-high and high BCd groups, respectively. Women had significantly higher BCd levels than those in men (0.97 versus 0.82 μg/L, *p* < 0.0001).

Trends toward increased BCd levels were seen with increasing age (P for trend <0.0001), daily smoking amount (P for trend <0.0001), education level (P for trend <0.0001), alcohol consumption (P for trend <0.0001), and regular walking (P for trend = 0.0003). Current smokers had a significantly higher BCd level than that in non-smokers (1.09 versus 0.85, *p* < 0.0001). Hypertensive patients had a significantly higher BCd level compared to that in patients without the corresponding disease (reference group) (1.10 versus 0.83, *p* < 0.0001). However, BCd levels did not differ significantly in subjects with stroke and IHD compared to those in the reference group.

### 3.2. Related Factor with High Blood Cadmium

**Figure 2:**
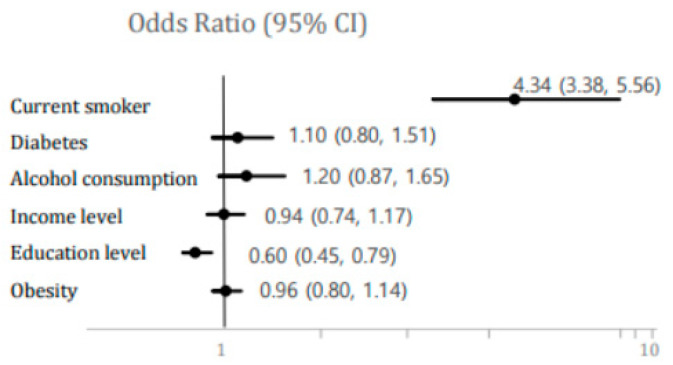
Odds ratios (ORs) (95% confidence intervals (CIs)) for high BCd. BCd levels were defined as follows: High BCd: >1.874 μg/L (90th percentile of BCd levels). References for each predictor: smoking status = never-smokers, diabetes mellitus = normal, alcohol consumption = none, income level = quartile 1, education level = less than high school diploma, obesity = normal.

**Figure 3:**
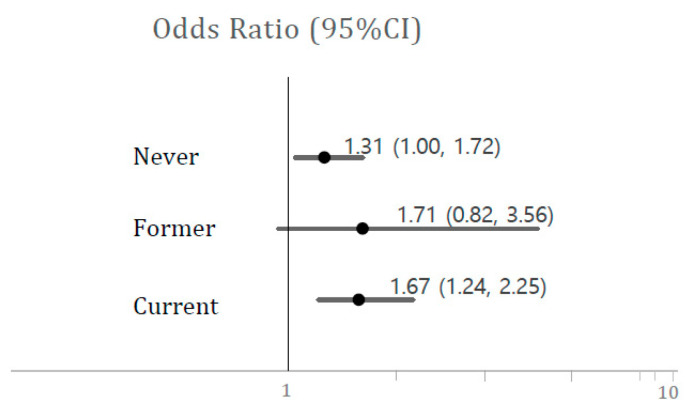
Odds ratios (95% CIs) of hypertension associated with high BCd level by smoking status. BCd levels were defined as follows: High BCd: >1.874 μg/L (90th percentile of BCd level). Associations between high BCd level and risks of stroke, IHD, and hypertension.

We assessed what factors were related to high BCd in the present study. Current smoking was a major related factor with high BCd (OR, 4.34; 95% CI, 3.38–5.56) (Figure 2). Thus, we analyzed the association between BCd and prevalent hypertension according to smoking status because current smoking was related to high BCd (Figure 3). However, there was no interaction between smoking and BCd (*p* = 0.9430, data not shown). Cadmium levels were related to higher risks of prevalent hypertension in both never and current smokers but the risk was higher in current smokers (never-smokers, OR, 1.31; 95% CI, 1.00–1.72; current smokers, OR; 1.67, 95% CI, 1.24–2.25) (Figure 3).

### 3.3. Associations between High BCd Level and Risks of Stroke, IHD, and Hypertension

The odds ratios (ORs) and 95% confidence intervals (CIs) for stroke, hypertension, and IHD are shown in Table 3. Previous studies reported that renal dysfunction was related to cadmium accumulation [43,44]. Thus, we further adjusted eGFR in a survey logistic regression model. Based on Model 2, the multivariate logistic regression model showed that high BCd level (>90th percentile) increased the risk of prevalent stroke and hypertension (stroke, OR, 2.39; 95% CI, 1.03–5.56; hypertension, OR, 1.46; 95% CI, 1.20–1.76). The association between high BCd hypertension was significant in both sexes (men, OR, 1.39; 95% CI, 1.07–1.80; women, OR, 1.45; 95% CI, 1.10–1.90) (data not shown). The risk of prevalent IHD was not associated with high BCd level. After adjusting, the changes of systolic and diastolic blood pressures in the high BCd level group were 3.00 (95% CI, 1.82–4.17) and 1.92 (95% CI, 1.14–2.70) mmHg, respectively (Table 4).

## 4. Discussion

The results of the present study showed the distributions of BCd levels according to the risk factors for prevalent CVD and the associations between high BCd levels and the risks of prevalent stroke, hypertension, and IHD among the Korean population under 60 years of age. In our study, high BCd level was related to higher risks of stroke and hypertension significantly. The association between high BCd level and prevalence of hypertension was shown in both current smokers and never-smokers but it was stronger in current smokers. There was no association between high BCd level and prevalent IHD risk.

The health effects of cadmium exposure have been reported as increased risks of CVD, atherosclerosis [45], stroke [46,47], high blood pressure, hypertension [32,48], and cancer [21]. Among these diseases, the results of several experimental studies support the effect of cadmium toxicity on CVD risk, including endothelial cell death, oxidative stress, smooth muscle cell accumulation, and vascular inflammation [27,28]. After cadmium is absorbed through the respiratory and digestive tracts, it is transported to the blood bound to metallothionein, which is important for cadmium metabolism and toxicokinetics [49,50]. Cadmium toxicity reduces glutathione and alters sulfhydryl homeostasis [51], which elevates oxidative stress indirectly and has led to increased blood pressure in rat models [52]. Increased oxidative stress may affect nitric oxide (NO), which is important for the regulation and modulation of cerebral blood flow, neuronal activity, and thrombogenesis [53]. Cadmium in blood increased oxidative stress, which is related to high blood pressure [52] and vascular injury [54].

As previous studies have defined the cut-off point for high BCd as the 90th percentile in the non-elderly population [39,55], we also used this cut-off point in our study to assess the cadmium effect for CVD risks among non-elderly individuals. Specifically, the US Environmental Protection Agency (USEPA) reported an advisory level of BCd concentration to be 1.7 µg/L based on human studies for proteinuria that suggested the oral reference dose (RfD) value to calculate the advisory level of BCd [56]. In the present study, the quartile-based analysis of BCd was not shown because the 75th percentile (1.348 µg/L) was considerably lower than the value of 1.7 µg/L as the cut-off value of BCd stated in the USEPA guidelines.

Our study showed a higher association between high BCd and stroke events in non-elderly individuals (<60 years). Although a previous Korean study among individuals ≥20 years [30] and a meta-analysis of overall adults and middle-aged to elderly populations suggested that cadmium level was not associated with the risk of stroke [29], a Chinese case–control study reported that BCd level was significantly related to the risk of ischemic stroke (IS) among those under 60 years of age (OR, 4.58; 95% CI, 2.82–7.45) [47]. Our results and those of the Chinese study showed that high cadmium levels might be associated with the risk of stroke in non-elderly individuals.

In our study, high BCd level was related to the prevalence of hypertension among participants under 60 years of age, a finding consistent with those of other Korean studies using KNHANES data from adults ≥20 years of age [30,31,32,33]. In Korea, high BCd levels might be strongly related to the risk of hypertension regardless of age. However, there were no association between cadmium level and hypertension risk in adults aged ≥50 years and aged ≥20 years, respectively, in a Japanese [57] and a US study [48]. There was a limitation to comparing results about Cd–hypertension association because the participant characteristics of each study were different. Thus, further study will be needed to find environmental or genetic effects of BCd for hypertension considering ethnic background.

A meta-analysis, including studies evaluating adults aged ≥20 years, middle-aged adults, and elderly adults, suggested that cadmium was associated with coronary heart disease (CHD), including myocardial infarction (MI) and IHD (pooled relative risk (RR), 1.30; 95% CI, 1.12–1.52) [29]. A prospective observational cohort study suggested that BCd was associated with the incidence of heart failure [58]. In contrast, we found that a high BCd level was not related to the risk of IHD in individuals under 60 years of age. To our knowledge, no other study has assessed the relationship between cadmium concentration and IHD risk in non-elderly individuals. Further study with more cases of IHD is required to verify the effect of cadmium on IHD risk in non-elderly individuals.

We assessed the effect of BCd levels on the risk of hypertension according to smoking status because smoking is an important determinant of cadmium exposure [21,22] as well as a major cause of CVD [59]. In our subgroup analysis, both never and current smokers with high BCd levels had higher risks of hypertension but the risk was stronger among current smokers. A Chinese study assessed the association between cadmium level and ischemic stroke (IS) events according to cigarette smoking status [47], reporting a higher risk of IS among current smokers than that among never-smokers. As tobacco smoke contains toxic components such as banzo[α]pyrene and nicotine, it may affect cadmium-induced inflammation and oxidative damage [60,61], which may increase the risk of CVD [53,62]. We recently reported that cigarette smoke extract (CSE) and Cd might induce neuroinflammation [63].

The strength of the present study was using the KNHANES data, nationally representative samples of Korea. These data were collected with a well-defined protocol by KCDC and related academic societies. In addition, we adjusted potential confounders, particularly alcohol consumption, BMI, glucose level, SBP, and physical activity to reduce bias.

Our study had the following limitations. First, we could not find dose-dependent changes in the BCd–stroke/IHD/hypertension association. We divided BCd levels by quartile to assess dose-dependent changes but we could not find any. In a complex biological system with respect to a toxicant, dose–response relationships could not be observed because some chemicals increased the non-monotonic relationship [64]. Further study will be needed to consider the non-monotonic dose–response relationship. Second, the cross-sectional design of the KNHANES prevents the determination of causality between exposures and outcomes. Third, the use of self-reported questionnaires to define stroke and IHD events could lead to misclassification. Fourth, we only used BCd data because the KHANES did not include urine cadmium information. However, previous studies have suggested that BCd levels reflect both short- and long-term exposure cadmium [65]. Thus, both blood and urine cadmium levels can be used as cadmium biomarkers [29]. Fifth, the confounding effect of smoking may have remained. We could not use biomarkers of smoking such as nicotine and cotinine. The nicotine level was not measured in the KNHANES survey and cotinine was not measured in each of the 2008–2013 and 2016 years. Finally, we could not classify the data by stroke subtype such as ischemic and hemorrhagic as this information was not included in the KNHANES data. However, almost all stroke patients in the present study likely experienced ischemic stroke because its incidence rate is the highest in Korea according to data from the 5th (2013) and 6th (2014) nationwide Acute Stroke Quality Assessment Program (ASQAP) [15].

## 5. Conclusions

In conclusion, this study is one of the few ones to identify an association between BCd and prevalent CVD risk in a non-elderly population. The results of the present study suggest that high BCd level is associated with an increased risk of prevalent stroke and hypertension in individuals aged 20–59 years. Further studies are required to assess the reasons for this association between cadmium and stroke and hypertension in non-elderly individuals.

## Figures and Tables

**Table 1:** General participant characteristics according to blood cadmium (BCd) level ^a.^

	Total (*n* = 10,626)		Non-High BCd (*n* = 9565)		High BCd (*n* = 1061)		*p*-Value ^b^
**Variables**	**Mean ± SD**		**Mean ± SD**		**Mean ± SD**		
Age (years)	40.14 ± 11.19		39.37 ± 11.17		47.12 ± 8.74		<0.0001
Age	*n*	weighted % (SE)	*n*	weighted % (SE)	*n*	weighted % (SE)	
20–29	2426	22.81 (0.43)	2369	24.66 (0.47)	57	5.83 (0.89)	<0.0001
30–39	2711	26.27 (0.43)	2564	27.54 (0.47)	147	14.62 (1.29)	
40–49	2740	27.66 (0.39)	2370	26.51 (0.43)	370	38.22 (1.68)	
50–59	2749	23.26 (0.37)	2262	21.29 (0.39)	487	41.33 (1.69)	
Sex							
Male	5081	50.37 (0.38)	4702	51.71 (0.42)	379	38.10 (1.69)	<0.0001
Female	5545	49.63 (0.38)	4863	48.29 (0.42)	682	61.90 (1.69)	
Smoking status							
Never	5947	54.41 (0.49)	5365	54.56 (0.52)	582	53.11 (1.82)	<0.0001
Former	1914	18.36 (0.43)	1841	19.61 (0.47)	73	6.87 (0.90)	
Current	2765	27.23 (0.50)	2359	25.83 (0.52)	406	40.03 (1.79)	
Daily smoking amount (the number of cigarettes)	3.93 ± 7.90		3.60 ± 7.48		6.94 ± 10.54		<0.0001
Education							
Less than high school diploma	1848	17.11 (0.44)	1471	15.31 (0.43)	377	33.59 (1.74)	<0.0001
High school diploma	4386	43.21 (0.61)	3944	42.99 (0.64)	442	45.21 (1.88)	
College diploma or higher	4392	39.68 (0.63)	4150	41.70 (0.66)	242	21.20 (1.51)	
Household income							
Quartile 1	2668	26.21 (0.59)	2337	25.64 (0.60)	331	31.51 (1.67)	0.0003
Quartile 2	2672	25.00 (0.53)	2392	24.88 (0.55)	280	26.04 (1.52)	
Quartile 3	2607	24.62 (0.53)	2375	24.82 (0.56)	232	22.86 (1.47)	
Quartile 4	2679	24.17 (0.60)	2461	24.67 (0.63)	218	19.60 (1.43)	
Frequency of alcohol drinks per week							
0	1994	18.13 (0.44)	1725	17.44 (0.45)	269	24.49 (1.58)	<0.0001
1	6094	57.47 (0.57)	5619	58.85 (0.59)	475	44.86 (1.85)	
2 or 3	1845	17.57 (0.42)	1650	17.53 (0.44)	195	17.94 (1.46)	
≥4	693	6.82 (0.30)	571	6.18 (0.30)	122	12.71 (1.25)	
LDL cholesterol (mg/dL)	113.26 ± 31.54		112.99 ± 31.15		115.62 ± 34.89		0.2330
Fasting glucose level (mg/dL)	95.58 ± 20.58		95.24 ± 20.28		98.70 ± 22.86		<0.0001
Estimated glomerular filtration rate (eGFR, mL/min/1.73 m^2^)	97.67 ± 17.27		97.91 ± 17.14		95.50 ± 18.20		0.0149
SBP (mmHg)	114.66 ± 15.06		114.06 ± 14.71		120.02 ± 16.98		<0.0001
DBP (mmHg)	76.24 ± 10.81		75.91 ± 10.69		79.19 ± 11.48		<0.0001
BMI (kg/m^2^)	23.63 ± 3.45		23.59 ± 3.46		23.95 ± 3.36		0.0028
Obesity ^c^							
No	7278	67.94 (0.56)	6569	68.18 (0.58)	709	65.81 (1.76)	0.1887
Yes	3348	32.06 (0.56)	2996	31.82 (0.58)	352	34.19 (1.76)	
Regular walking ^d^							
No	6271	58.64 (0.57)	5607	58.11 (0.60)	664	63.45 (1.77)	0.0049
Yes	4355	41.36 (0.57)	3958	41.89 (0.60)	397	36.55 (1.77)	
Diabetes mellitus							
No	8151	76.83 (0.51)	7435	77.93 (0.53)	716	66.71 (1.69)	<0.0001
Impaired fasting glucose	1877	17.62 (0.45)	1613	16.80 (0.47)	264	25.17 (1.61)	
Yes	598	5.55 (0.25)	517	5.27 (0.26)	81	8.12 (1.00)	
Stroke							
No	10,566	99.54 (0.07)	9517	99.60 (0.07)	1049	98.99 (0.36)	0.0141
Yes	60	0.46 (0.07)	48	0.40 (0.07)	12	1.01 (0.36)	
Hypertension							
No	6108	57.30 (0.62)	5640	58.72 (0.62)	468	44.23 (1.84)	<0.0001
Pre-hypertension	2588	24.41 (0.50)	2318	24.31 (0.53)	270	25.31 (1.67)	
Yes	1930	18.30 (0.46)	1607	16.97 (0.46)	323	30.46 (1.64)	
Ischemic heart disease							
No	10,546	99.25 (0.10)	9501	99.31 (0.10)	1045	98.73 (0.39)	0.0766
Yes	80	0.75 (0.09)	64	0.69 (0.10)	16	1.27 (0.39)	

SD, standard deviation; SE, standard error; BCd, blood cadmium; SBP, systolic blood pressure; DBP, diastolic blood pressure. ^a^ BCd levels were defined as follows: Non-high BCd: ≤ 1.874 μg/L (90th percentile of BCd level); High BCd: >1.874 μg/L. ^b^. *p*-value for the differences in frequency of each variable between the non-high and high BCd groups from the survey *t*-tests and Rao–Scott chi-square tests. ^c^ Obesity: body mass index (BMI) ≥ 25 kg/m^2^. ^d^ Regular walking: walking at least 30 min at a time and ≥5 days a week during the last seven days.

**Table 2:** Distributions of cadmium levels (μg/L) of general and clinical characteristics according to BCd level ^a^.

	Total (*n* = 10,626)	Non-High BCd (*n* = 9565)	High BCd (*n* = 1061)
**Variables**	**Geometric mean (SE)**	**Geometric mean (SE)**	**Geometric mean (SE)**
Total	0.89 (0.01)	0.80 (0.01)	2.40 (0.02) **
Age (years)			
20–29	0.57 (0.01)	0.55 (0.01)	2.34 (0.09)
30–39	0.83 (0.01)	0.78 (0.01)	2.35 (0.03)
40–49	1.06 (0.01)	0.93 (0.01)	2.41 (0.03)
50–59	1.20 (0.01)	1.04 (0.01)	2.42 (0.03)
P for trend ^b^	<0.0001	<0.0001	0.7767
Sex			
Male	0.82 (0.01)	0.75 (0.01)	2.37 (0.03)
Female	0.97 (0.01) **	0.85 (0.01) **	2.42 (0.02)
Smoking status			
Never	0.85 (0.01)	0.76 (0.01)	2.43 (0.03)
Former	0.75 (0.01)	0.72 (0.01) *	2.22 (0.04) *
Current	1.09 (0.01) **	0.96 (0.01) **	2.39 (0.03)
Daily smoking amount (the number of cigarettes)			
<10	0.83 (0.01)	0.75 (0.01)	2.41 (0.02)
10–14	1.00 (0.02)	0.91 (0.02)	2.33 (0.02)
15–19	1.10 (0.03)	0.98 (0.02)	2.46 (0.10)
20–70	1.30 (0.02)	1.11 (0.02)	2.39 (0.04)
P for trend ^b^	<0.0001	<0.0001	0.2773
Education			
Less than high school diploma	1.24 (0.02)	1.06 (0.01)	2.40 (0.04)
High school diploma	0.89 (0.01)	0.79 (0.01)	2.43 (0.03)
College diploma or higher	0.77 (0.01)	0.72 (0.01)	2.34 (0.03)
P for trend	<0.0001	<0.0001	0.0694
Household income			
Quartile 1	0.95 (0.01)	0.83 (0.01)	2.43 (0.03)
Quartile 2	0.91 (0.01)	0.81 (0.01)	2.38 (0.04)
Quartile 3	0.87 (0.01)	0.79 (0.01)	2.39 (0.04)
Quartile 4	0.83 (0.01)	0.76 (0.01)	2.40 (0.04)
P for trend	0.5947	0.7332	0.6763
Frequency of alcohol consumption per week			
0	0.96 (0.02)	0.83 (0.01)	2.42 (0.04)
1	0.83 (0.01)	0.76 (0.01)	2.37 (0.03)
2 or 3	0.94 (0.02)	0.85 (0.01)	2.39 (0.04)
≥4	1.19 (0.03)	1.01 (0.02)	2.48 (0.07)
P for trend	<0.0001	<0.0001	0.4335
Obesity ^c^			
No	0.88 (0.01)	0.79 (0.01)	2.41 (0.02)
Yes	0.92 (0.01)	0.82 (0.01)	2.39 (0.03)
Regular walking ^d^ (n, weighted %)			
No	0.93 (0.01)	0.83 (0.01)	2.40 (0.02)
Yes	0.84 (0.01)	0.76 (0.01)	2.40 (0.03)
P for trend	0.0003	0.0007	0.6745
Diabetes mellitus			
No	0.85 (0.01)	0.77 (0.01)	2.40 (0.02)
Impaired fasting glucose	1.02 (0.02)	0.89 (0.01)	2.41 (0.04)
Yes	1.05 (0.03)	0.92 (0.02)	2.40 (0.08)
Stroke			
No	0.89 (0.01)	0.80 (0.01)	2.40 (0.02)
Yes	0.94 (0.07)	0.74 (0.04) *	2.28 (0.04)
Hypertension			
No	0.83 (0.01)	0.76 (0.01)	2.33 (0.02)
Pre-hypertension	0.90 (0.01) *	0.80 (0.01) *	2.46 (0.04) *
Yes	1.10 (0.02) **	0.94 (0.01) *	2.46 (0.04) *
Ischemic heart disease			
No	0.89 (0.01)	0.80 (0.01)	2.40 (0.02)
Yes	1.10 (0.06)	0.94 (0.04)	2.41 (0.09)

SE, standard error; CI, confidence interval; BCd, blood cadmium. ^a^ BCd levels were defined as follows: Non-high BCd: ≤1.874 μg/L (90th percentile of BCd levels); High BCd: >1.874 μg/L. ^b^ *p*-value for trend was assessed by survey regression test. Adjusted for age, sex, current smoking status, education level, income level, alcohol consumption, BMI, glucose level, systolic blood pressure, survey year, and regular walking. ^c^ Obesity: body mass index (BMI) ≥25 kg/m^2^. ^d^ Regular walking: walking at least 30 min at a time and ≥5 days a week during the last seven days.* *p* < 0.05, ** *p* < 0.0001 for the comparison geometric mean with the reference group for each variable (age 20–29 years, male sex, never-smoker, daily cigarettes < 10, education less than high school diploma, low income level, no alcohol consumption per week, obesity, regular walking, diabetes, stroke, hypertension, and ischemic heart disease.

**Table 3:** Odds ratios (95% CIs) of cardiovascular diseases associated with high BCd level ^a^.

		Total (*n* = 10,626)			
	Cases/Non-Cases	Crude OR (95% CI)	Model 1 ^b^ OR (95% CI)	Model 2 ^c^ OR (95% CI)	Model 3 ^d^ OR (95% CI)
Stroke					
Non-high BCd	48/9517	1.00 (reference)	1.00 (reference)	1.00 (reference)	1.00 (reference)
High BCd	12/1049	2.55 (1.18, 5.51)	2.37 (1.02, 5.49)	2.39 (1.03, 5.56)	2.50 (1.01, 6.18)
Hypertension					
Non-high BCd	1607/7958	1.00 (reference)	1.00 (reference)	1.00 (reference)	1.00 (reference)
High BCd	323/738	2.14 (1.83, 2.51)	1.45 (1.20, 1.76)	1.46 (1.20, 1.76)	1.48 (1.22, 1.79)
Ischemic heart disease					
Non-high BCd	64/9501	1.00 (reference)	1.00 (reference)	1.00 (reference)	1.00 (reference)
High BCd	16/1045	1.84 (0.93, 3.66)	1.11 (0.55, 2.24)	1.12 (0.56, 2.26)	1.08 (0.54, 2.15)

OR, odds ratio; CI, confidence interval; BCd, blood cadmium. ^a^ BCd levels were defined as follows: Non-high BCd: ≤1.874 μg/L (90th percentile of BCd levels); High BCd: >1.874 μg/L. ^b^ Model 1: Adjusted for age, sex, current smoking status, education level, income level, alcohol consumption, BMI, glucose level, systolic blood pressure (SBP), survey year, and regular walking (SBP was not adjusted for hypertension). ^c^ Model 2: Further adjusted for estimated glomerular filtration rate (eGFR, mL/min/1.73 m^2^). ^d^ Model 3: Adjusted for daily smoking amount (the number of cigarettes) instead of current smoking status in Model 1.

**Table 4:** Change (95% CIs) of systolic and diastolic blood pressure levels according to BCd level (μg/L).

		Systolic Blood Pressure (mmHg)		Diastolic Blood Pressure (mmHg)	
BCd level	*n*	Crude	Adjusted ^b^	Crude	Adjusted ^b^
BCd (μg/L)	10,626	4.35 (3.83, 4.87) **	2.08 (1.50, 2.66) *	2.67 (2.28, 3.05) **	1.39 (0.97, 1.81) *
Non-high BCd ^a^	9565	0.00 (reference)	0.00 (reference)	0.00 (reference)	0.00 (reference)
High BCd ^a^	1,061	5.85 (4.61, 7.10)	3.00 (1.82, 4.17)	3.33 (2.50, 4.16)	1.92 (1.14, 2.70)
P for trend ^c^		<0.0001	<0.0001	<0.0001	<0.0001

^a^ High BCd >90th percentile of BCd levels. Non-high BCd: ≤1.874 μg/L; High BCd: >1.874 μg/L. ^b^ Adjusted for age, sex, current smoking status, education level, income level, alcohol consumption, BMI, glucose level, survey year, and regular walking. ^c^ *p* for trend was assessed by survey regression test. * *p* < 0.05, ** *p* < 0.0001.
